# Multigene Profiling of Circulating Tumor Cells in Esophageal Squamous Cell Carcinoma Identifies Prognostic Cancer Driver Genes Associated with Epithelial-Mesenchymal-Transition Progression and Chemoresistance

**DOI:** 10.3390/cancers15225329

**Published:** 2023-11-08

**Authors:** Zhen Tan, Josephine Mun-Yee Ko, Valen Zhuoyou Yu, Ka-On Lam, Dora Lai-Wan Kwong, Ian Yu-Hong Wong, Fion Siu-Yin Chan, Claudia Lai-Yin Wong, Kwan-Kit Chan, Tsz-Ting Law, Faith Sin-Fai Choy, Hoi-Yan Ng, Simon Ying-Kit Law, Maria Li Lung

**Affiliations:** 1 Department of Clinical Oncology, School of Clinical Medicine, University of Hong Kong, Hong Kong, China; 2 Department of Surgery, School of Clinical Medicine, University of Hong Kong, Hong Kong, China

**Keywords:** CTC, EMT, Gene expression, *TWIST1*, ESCC, Xenograft

## Abstract

**Simple Summary:**

Esophageal squamous cell carcinoma (ESCC) is a malignancy characterized by high mortality and dismal quality of life. Circulating tumor cells (CTCs), considered precursors of distant metastasis, can be analyzed using a non-invasive liquid biopsy approach to identify patients at risk of cancer progression or recurrence. The current study employed an unbiased size-based CTC enrichment strategy in combination with quantitative reverse transcription polymerase chain reaction (RT-qPCR) to investigate gene transcripts as potential biomarkers in the bloodstream. We categorized 83.6% (46/55) of ESCC patients as CTC-positive, each displaying at least two detected markers. Furthermore, 50.9% (28/55) of ESCC patients were identified as CTC-high, showing at least five detected markers using a 10-gene CTC panel. The presence of specific markers, namely *TWIST1*, *VEGFC*, *CCND1*, and *TFRC*, was significantly associated with shorter survival of the patients, suggesting their prognostic values as liquid biopsy markers to guide clinical ESCC treatment decisions and enhance treatment efficacy.

**Abstract:**

We investigated the clinical significance of CTCs in cancer progression by detecting multiple cancer driver genes associated with epithelial-to-mesenchymal transition (EMT) at the transcript level. The 10-gene panel, comprising *CCND1*, *ECT2*, *EpCAM*, *FSCN1*, *KRT5*, *KRT18*, *MET*, *TFRC*, *TWIST1*, and *VEGFC*, was established for characterizing CTCs from mouse ESCC xenograft models and clinical ESCC peripheral blood (PB) samples. Correlations between gene expression in CTCs from PB samples (*n* = 77) and clinicopathological features in ESCC patients (*n* = 55) were examined. The presence of CTCs at baseline was significantly correlated with tumor size (*p* = 0.031). The CTC-high patients were significantly correlated with advanced cancer stages (*p* = 0.013) and distant metastasis (*p* = 0.029). High mRNA levels of *TWIST1* (Hazard Ratio (HR) = 5.44, *p* = 0.007), *VEGFC* (HR = 6.67, *p* < 0.001), *TFRC* (HR = 2.63, *p* = 0.034), and *EpCAM* (HR = 2.53, *p* = 0.041) at baseline were significantly associated with a shorter overall survival (OS) in ESCC patients. This study also revealed that *TWIST1* facilitates EMT and enhances malignant potential by promoting tumor migration, invasion, and cisplatin chemoresistance through the *TWIST1-TGFBI-ZEB1* axis in ESCC, highlighting the prognostic and therapeutic potential of *TWIST1* in clinical ESCC treatment.

## 1. Introduction

Esophageal cancer (EC) ranks as the ninth most prevalent cancer and the sixth leading cause of global cancer deaths [1], with esophageal squamous cell carcinoma (ESCC) being the most predominant pathological subtype and contributing to 85% of all EC cases in 2020 [2].

The ESCC five-year survival rate is far from satisfactory and under 20%, primarily attributed to late presentation and technical challenges for the detection of metastasis and effective treatment options available for metastatic ESCC [3,4]. At diagnosis, approximately 32% of EC patients have regional disease and 50% of patients have evidence of distant metastatic disease, which largely prevents early intervention of ESCC with curative treatments [5]. Currently, a lack of insight regarding the molecular mechanisms contributing to its metastatic progression remains a challenge.

**Figure 1:**
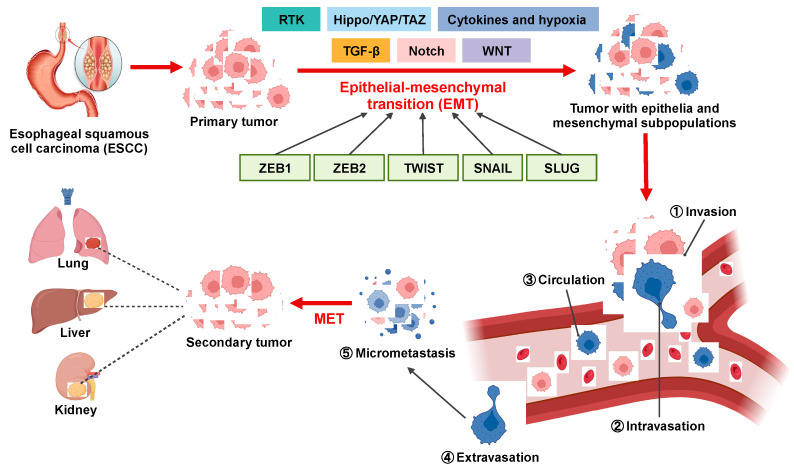
Overview of the involvement of EMT in ESCC metastatic cascade. EMT confers migratory and invasive potential to ESCC cells, enabling them to (1) locally invade the basement membrane, (2) intravasate into lymphatics and blood vessels, (3) circulate through lymphatics and blood vessels to distant tissues and organs, and (4) extravasate from lymphatics and blood vessels to (5) form micrometastases and secondary tumors.

Shedding from primary sites and dissemination into the bloodstream, circulating tumor cells (CTCs) are known to be associated with epithelial–mesenchymal-transition (EMT) and malignant progression of cancer cells [6,7] (Figure 1). These EMT-converted CTCs present in liquid biopsies could provide predictive or prognostic markers for monitoring cancer metastatic progression in cancer patients [8]. Despite numerous studies investigating CTCs in ESCC, the exact molecular mechanisms and intricacies of ESCC metastasis remain incompletely comprehended [9,10]. This is probably attributed to the exceptional scarcity and fragility of CTCs, as well as a deficiency in sensitive and reliable approaches for detection. Hence, innovative combined strategies for discovering and disseminating CTCs have the potential to pave the way for the development of novel therapeutic approaches for ESCC.

Previous CTC detection rates in ESCC using the CellSearch^®^ EpCAM-based selection approach range from 18% in surgically resectable cases to 28–50% in unresectable cases [11,12]. To mitigate potential biases stemming from EpCAM-based enrichment, non-immunomagnetic-based CTC technologies can be used to retrieve a more comprehensive population of CTCs for molecular subtyping [13,14]. The novel ClearCell^®^FX1 System enriches CTCs from blood samples using a microfluidic chip based on differential cancer cell size and deformability [13]. Based on this unbiased size-based enrichment strategy, various CTC subtypes can be identified, including epithelial, mesenchymal, and hybrid EMT phenotypes. Our previous studies using this platform demonstrated that continuous real-time monitoring of circulating tumor cells over time can offer valuable prognostic and predictive insights into treatment effectiveness, disease recurrence, and survival outcomes in advanced ESCC [15].

In this study, we hypothesized that molecular characterization of CTCs by sensitive qPCR assay with EMT property that facilitates their detachment from the primary tumor was useful to identify prognostic biomarkers for advanced ESCC patients. We successfully established CTC enrichment by size separation with a microfluidic device and a 10-gene panel to molecularly detect CTCs by mRNA profiling. Our longitudinal CTC monitoring findings demonstrated that high CTC phenotypes with high expression of *TWIST1*, *VEGFC*, *TFRC*, and *EpCAM* at baseline blood are unfavorable prognostic factors for survival in a cohort of 55 patients with locally advanced and metastatic ESCC. Further functional studies of *TWIST1* revealed its involvement in EMT progression and cisplatin chemoresistance in ESCC. In conclusion, our findings highlight *TWIST1*, as a sensitive biomarker for detecting CTCs and as a promising therapeutic target for the treatment of ESCC.

## 2. Materials and Methods

### 2.1. Cell Culture

ESCC cell lines, including KYSE-30 (Cellosaurus ID: CVCL_1351), KYSE70 (CVCL_1356), KYSE150 (CVCL_1348), KYSE180 (CVCL_1349), KYSE270 (CVCL_1350), KYSE450 (CVCL_1353), and KYSE510 (CVCL_1354) were cultured in a RPMI 1640 medium, supplemented with 10% fetal bovine serum (FBS), 1% penicillin-streptomycin (Invitrogen, Waltham, MA, USA) at 37 °C with 5% CO_2_. The cell lines were routinely tested and maintained negative for mycoplasma contamination.

### 2.2. Spike-in Experiments

The CTC recovery rate in spike-in experiments was routinely monitored using two different-sized ESCC cell lines (KYSE30 and KYSE270) in 7.5 mL of healthy blood, as previously described [10]. The mean recovery rate for each cell line (~200 cells) was 73.43 ± 8% for KYSE30 (average size ~17 μm) and 62 ± 10% for KYSE270 (average size ~15 μm), ensuring reproducibility of the CTC enrichment. With the 10× Chromium Single Cell Gene Expression assay for exploring transcriptomic cellular heterogeneity, a total of 200 KYSE270 cells were identified and spiked into 7.5 mL healthy blood, followed by CTC enrichment with ClearCell^®^FX1 system (Biolidics, Singapore), library preparation (Chromium Single Cell 3’ v2 Reagent Kit, 10× Genomics^®^, Pleasanton, CA, USA) and sequencing as previously described [16]. For the RT-qPCR assay, KYSE30 and KYSE270 cells were directly spiked into 50,000 peripheral blood mononuclear cells (PBMCs) at ratios ranging from 1:100 to 1:10,000 without prior enrichment, followed by further RNA extraction.

### 2.3. Patient and Sample Collection

A total of 55 ESCC patients and 4 colorectal cancer (CRC) patients at Queen Mary Hospital between 2020 and 2023 were studied. The enrolled ESCC patients were categorized into two groups: the palliative treatment group (Palliative CT/CTRT, *n* = 24) and the curative treatment group (Curative CTRT/upfront surgery/Neoadjuvant CT + surgery, *n* = 31), based on their clinical treatment history. A total of 81 PB samples (ESCC = 77, CRC = 4) were collected from these 59 patients. The timeline for collecting PB specimens for CTC detection included a baseline time point taken before treatment for each patient [10,15]. For patients, who received palliative treatments, the pre-III time point was captured after two CT cycles. For patients undergoing curative treatments, CTC detection was conducted one month after surgery. Consent for the collection of samples from ESCC patients was obtained following protocols approved by the Institutional Review Board (IRB) of the University of Hong Kong/Hospital Authority Hong Kong West Cluster (HKU/HA HKW IRB). The study was conducted in compliance with the principles of the Declaration of Helsinki.

### 2.4. CTC Enrichment, RNA Extraction and RT-qPCR

PB samples (7.5 mL) were collected using VACUETTE^®^ EDTA tubes and processed for CTC enrichment using CTChip^®^FR1 microfluidic chips (ClearCell^®^FX1 System, Biolidics, Singapore), as previously described [15,17]. Total RNA was isolated using the TRIzol^TM^ Reagent (Invitrogen, Carlsbad, CA, USA) as per the manufacturer’s instructions. Complementary DNA was synthesized from total RNA using SuperScript^®^ IV Reverse Transcriptase (Invitrogen, CA, USA) with oligo-dT (Promega, Madison, WI, USA) under the following conditions: 65 °C for 5 min, 55 °C for 10 min, 80 °C for 10 min, and then held at 4 °C. The transcript level of target genes (*n* = 44) was determined using a two-step quantitative PCR technique with FastStart^TM^ Universal SYBR Master (Rox) (Roche, Basel, Switzerland) on the LightCycler^®^ 480 System (Roche Diagnostics, Basel, Switzerland), following the manufacturer’s instructions. The 2^−ΔΔCt^ method was used to calculate fold changes normalized to *GAPDH*, with genes considered positively expressed at fold change values exceeding 2. All primers used in this study for RT-qPCR are listed in Table S1.

### 2.5. Lentivirus Preparation and Transfection

Clustered Regularly Interspaced Short Palindromic Repeat (CRISPR) systems were used with gene coding sequences (*TWIST1*, *TGFBI*, *ZEB1*) and sgRNA oligos (*TWIST1*) to generate overexpression and knockdown cell lines, respectively [18]. Lentivirus preparation and infection were performed as described. Primers used for gene cloning and knockdown are listed in Tables S2 and S3.

### 2.6. RNA Sequencing and Transcriptomic Analysis

Total RNA from *GFP*- and *TWIST1*-expressing KYSE450 cells were extracted using the AllPrep^®^ DNA/RNA Micro Kit (Qiagen, Hilden, Germany), following the manufacturer’s instructions. Qualified samples were subjected to ribo-depleted RNA sequencing on the NovaSeq 6000 platform (Illumina, San Diego, CA, USA) at the HKU Centre for PanorOmic Sciences. Transcriptome analysis was conducted using the Partek^®^ Flow^®^ software (Version 11.0.23.1023) (Partek Incorporated, Chesterfield, MO, USA), as previously described [19]. Comprehensive gene set enrichment analysis downstream of *TWIST1* was performed using EnrichR and the Molecular Signatures Database (MSigDB) Hallmark 2020.

### 2.7. Western Blot Analysis

Cell protein lysates of KYSE150 and KYSE450 cells were electrophoresed on 8% SDS-PAGE gels. Proteins were transferred to PVDF membranes (Millipore, Billerica, MA, USA), blocked with 5% bovine serum albumin (BSA), and incubated with primary antibodies, as previously described. Detailed information for the antibodies used in this study is summarized in Table S4.

### 2.8. Wound Healing Assay

KYSE150 and KYSE450 cells (1 × 10^6^/well) were seeded into 6-well plates to form a confluent monolayer. Wounds were created by gently scratching cells using a 200 μL pipette tip, followed by incubation with fresh culture medium for 24 h. Images were captured using NIS-Elements software (Nikon, Tokyo, Japan) and the closure area was quantitatively assessed with ImageJ (version 1.53 h) (National Institutes of Health, Bethesda, MD, USA).

### 2.9. Matrigel-Coated Transwell Assay

KYSE150 and KYSE450 cells (2 × 10^5^/well) in a serum-free medium were loaded into the upper well of Matrigel-coated Transwell chambers (Corning Inc., Corning, NY, USA) for 24 h, with the lower well filled with a culture medium as the chemoattractant. Migrated cells in the lower compartments were stained with 1% crystal violet (Sigma-Aldrich, Saint Louis, MO, USA) following the manufacturer’s instructions. Images were captured by the Cytation 5 Cell Imaging Multi-Mode Reader (BioTek, Winooski, VT, USA) and the migrated cells were counted in 5 microscopic fields per well to calculate the average value.

### 2.10. Colony Formation Assay

KYSE150 and KYSE450 cells (2 × 10^3^/well) were seeded in 6-well plates for a 14-day culture. Cell colonies were stained with 1 × Giemsa (Sigma Aldrich, Saint Louis, MO, USA), as previously described [18].

### 2.11. MTT Assay Following Cytotoxic Drug Treatments

KYSE70, KYSE150, and KYSE450 cells (4 × 10^3^/well) were seeded in 96-well cell culture microplates in triplicate for 24 h to facilitate their exponential growth, followed by incubation with cisplatin (10 or 20 μM) in a fresh culture medium for an additional 24, 48 and 72 h. Cell proliferation and viability were assessed using the 3-(4,5-dimethylthiazol-2-yl)-2,5-diphenyltetrazolium bromide (MTT) assay as previously described [18].

### 2.12. In Vivo Tumorigenicity and In Vivo Metastasis Assay

Female BALB/c athymic nude mice (6–8 weeks old) were used for both subcutaneous patient-derived organoid xenograft (PDOX) models and in vivo metastasis models and performed as previously described [18]. For PDOX models, 2 × 10^6^ PDO cells/site were subcutaneously transplanted into both sides of each mouse (3 mice per group, with 3 healthy mice as controls). The ESCC PDOs used in this study were established as previously described [20]. For cardiac blood collection of ESCC PDOX models, the retrograde cardiac puncture method was performed in mice one month after tumor inoculation. Prior to cardiac puncture, each mouse was anesthetized, followed by insertion into the left ventricle with a 27G Microfine needle (BD Biosciences, Franklin Lakes, NJ, USA). Cardiac blood was drawn out through the needle using a syringe and then transferred to a VACUETTE^®^ EDTA tube (Greiner Bio-One, Frickenhausen, Germany) for subsequent CTC enrichment. For in vivo metastasis models, 1 × 10^6^ *GFP*- or *TWIST1*-overexpressing KYSE150Luc cells were transplanted into each mouse (8 mice per group) via tail-vein injection. Lung metastasis was monitored at 3- and 6-weeks post-injection using the IVIS^®^ Lumina^TM^ X5 Imaging System (PerkinElmer, Waltham, MA, USA). All animal experimentation was conducted in accordance with the protocols endorsed by the Committee on the Use of Live Animals in Teaching and Research of the University of Hong Kong (CULATR NO.6006-22).

### 2.13. Statistics

The Kolmogorov–Smirnov non-parametric test was used to compare the differences in fold change between CTC markers in the healthy donor (HD) groups and the CTC groups.

Fisher’s exact tests and Pearson chi-square tests were used to assess the association between CTC marker positivity and categorical clinicopathological factors. CTC status was categorized into a CTC-positive group (≥2 detected markers) or CTC-high group (≥5 detected markers) with a threshold determined using a receiver operating characteristic (ROC) curve analysis for assessing progression-free survival (PFS) and OS (Tables S5 and S6). PFS or OS were defined by the period between treatment start to date of clinical progression or date of death, respectively. Patients were censored at the last follow-up (31 May 2023) or death. The median follow-up was 4 months for palliative patients and 10 months for curative patients. CTC samples showing the co-expression of *EpCAM* and *TWIST1* mRNA were classified as hybrid EMT. The Kaplan–Meier analysis was used to compare both PFS and OS with marker positivity or negativity. The Cox regression models with gender, age, and TNM stages as covariates were used to calculate hazard ratios (HRs) for OS. Student’s *t*-test was used for all statistical analyses of functional studies. A significance level of *p*-value < 0.05 (two-sided) was used for all analyses. Survival analyses were performed using SPSS (version: 28.0.1.0), and GraphPad PRISM (version: 9.5.1) was used for graphic design and analysis. The EMT schematic illustration was created using Biorender.com, accessed on 13 October 2023.

## 3. Results

### 3.1. Establishment of Gene Panels for CTC Detection in ESCC Patients

**Figure 2:**
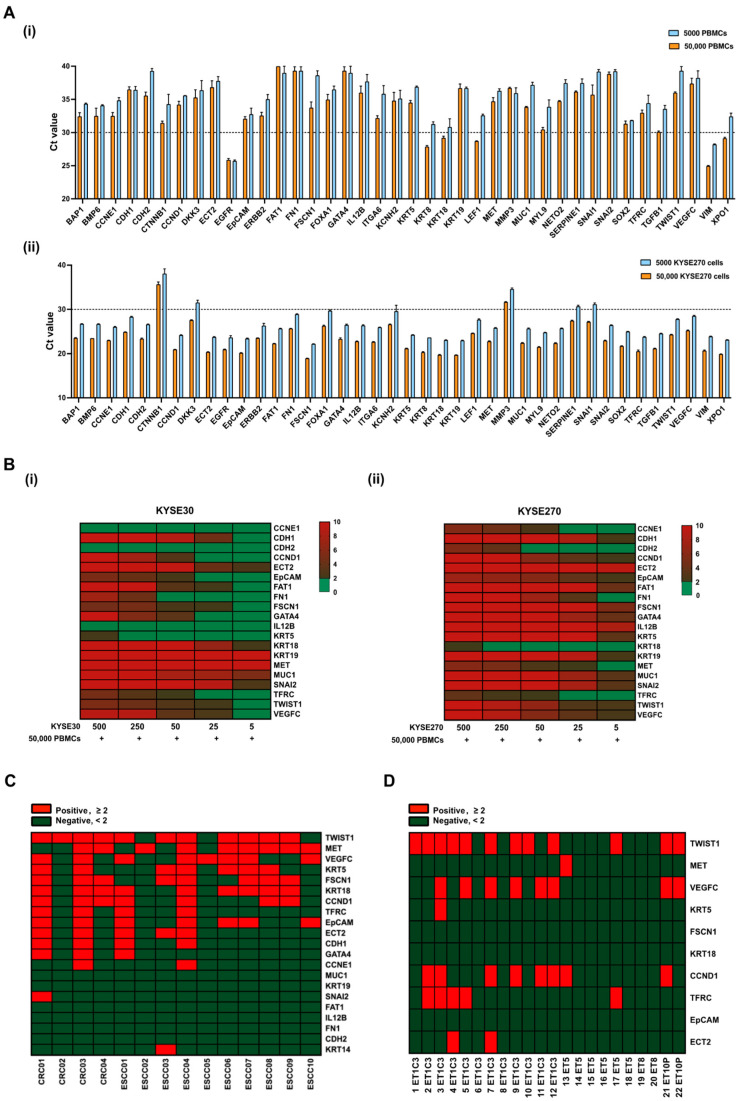
Establishment of gene panels for CTC detection in patients with ESCC. (**A**) Transcript levels of 40 CTC markers in (**i**) PBMCs, and (**ii**) KYSE270 cells were detected by RT-qPCR and indicated with Ct values; error bars represent standard error mean. (**B**) Spike-in assay with (**i**) KYSE30/PBMCs, and (**ii**) KYSE270/PBMCs was performed to determine the sensitivity of 20 CTC markers in ESCC using RT-qPCR. Control, 50,000 PBMCs. (**C**) Heatmap of transcript levels of 20 CTC markers in CTCs from a pilot study of 14 clinical PB samples by RT-qPCR. Control, HDs. CRC, colorectal cancer. (**D**) Heatmap of transcript levels of 10 CTC markers in CTCs from cardiac blood of ESCC PDOX mice by RT-qPCR. Control, healthy nude mice.

The isolation and identification of CTCs in ESCC patients are crucial for early cancer diagnosis and prognosis. Liquid biopsy-based CTC detection, involving RT-qPCR analysis of blood samples, provides a feasible approach to identifying metastatic cancer cells prior to clinical detection. Nevertheless, CTCs remain scarce and can potentially retain leukocyte contamination even after enrichment. To minimize background noise, optimal CTC markers should be selectively enriched in cancer cells and not expressed in leukocytes. Initially, a set of 224 genes related to EMT and tumor metastasis were screened using an ESCC cell spike-in assay alongside peripheral blood (PB) samples from a healthy donor (HD) for a 10× Chromium single-cell gene expression analysis (Table S7). Nearly half of the KYSE270 cells (~105) were recovered and identified using the epithelial marker genes, including *EpCAM*, *KRT8*, *KRT18*, and *KRT19*, from the initial 200 spiked KYSE270 cells in the 10× spike-in assay. Based on bioinformatic analysis of the sequencing data (average reads in peripheral blood mononuclear cells (PBMCs) < 0.10, average reads in KYSE270 > 1.0), 40 CTC marker genes were chosen. Transcript levels of these genes in 50 single KYSE270 cells are shown in a heatmap to reveal intratumor heterogeneity (Figure S1). Further RT-qPCR validation of these genes was conducted in PBMCs and KYSE270 cells (Figure 2A). Genes enriched in PBMCs (Ct value < 30) or lowly expressed in ESCC cells (Ct value > 30) were excluded (*n* = 12) from subsequent spike-in assays; these include *EGFR*, *KRT8*, *LEF1*, *MYL9*, *TGFB1*, *VIM*, *XPO1*, *CTNNB1*, *DKK3*, *MMP3*, *SERPINE1*, and *SNAI1*. To encompass a broad spectrum of cancer signatures, the high mesenchymal KYSE270 cells and the high epithelial KYSE30 cells were chosen for the subsequent spike-in assay based on scores of generic EMT signatures (Figure S2). A total of 28 genes were enrolled for the RT-qPCR spike-in assay with corresponding ESCC cells and 50,000 PBMCs. Transcripts of 20 genes were positively detected when spiked with 500 ESCC cells (both KYSE30 and KYSE270) (Figure 2B). To further assess the potential of these markers in characterizing clinical CTCs, 20 selected CTC markers from the spike-in assay were employed in a pilot study involving 14 clinical PB samples. Distinct sensitivities of these markers were identified and are shown in the heatmap (Figure 2C). Markers with an overall positivity rate below 30% in the pilot study were excluded from subsequent clinical CTC characterization, including *CDH1*, *GATA4*, *CCNE1*, *MUC1*, *KRT19*, *SNAI2*, *FAT1*, *IL12B*, *FN1*, *CDH2* and *KRT14*. In addition to the pilot study involving clinical PB samples, CTCs were also enriched from the cardiac blood of ESCC PDOX mouse models, including ET1C3, ET5, ET8, and ET10P. Among the 10-gene panel, *TWIST1*, *CCND1*, and *VEGFC* emerged as the most sensitive markers for ESCC PDOX CTC characterization (Figure 2D).

### 3.2. Evaluation of Gene Panels for Characterizing CTCs in ESCC Patients

**Figure 3:**
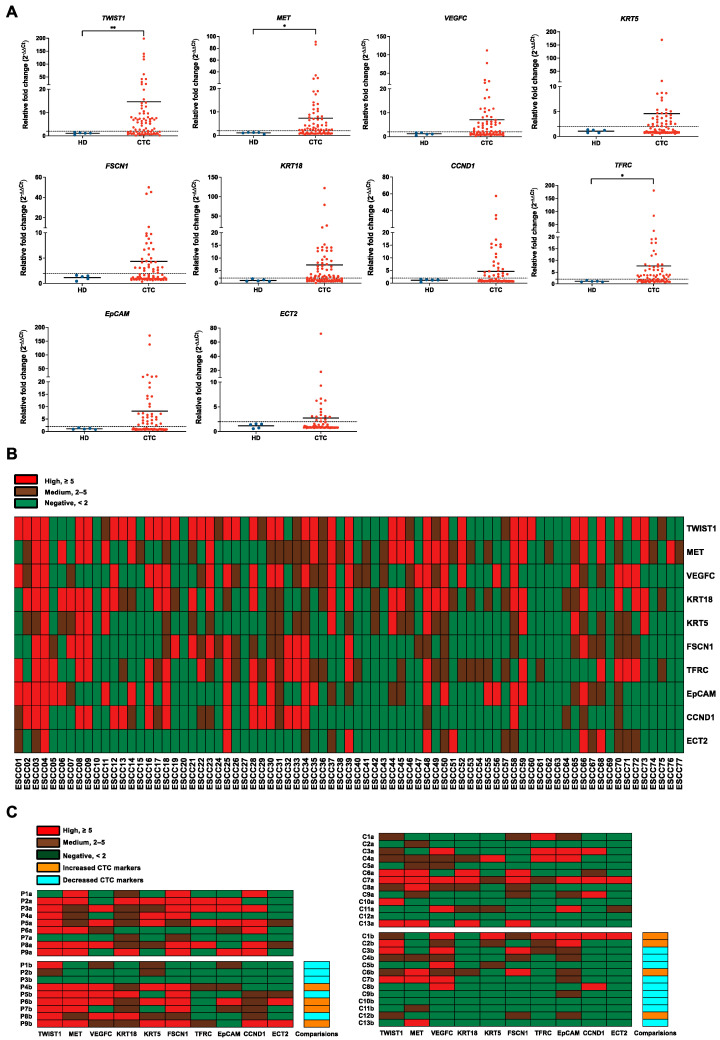
Evaluation of gene panels for characterizing CTCs in patients with ESCC. (**A**) Gene expression correction using RT-qPCR for leukocyte contamination in CTC samples. The data were visualized as a scatter plot with the median, and the line represents the reference normal fold change value. HDs (*n* = 5), CTC (*n* = 77). ** *p*-value < 0.01, * *p*-value < 0.05. (**B**) Heatmap of transcript levels of 10 CTC markers in 77 ESCC samples by RT-qPCR. Control, HDs. (**C**) Heatmap of 10 CTC markers in 22 pairs of ESCC samples between different clinical time points using RT-qPCR. P, palliative treatment, *n* = 9; C, curative treatment, *n* = 13; a, baseline; b, pre-III or one-month post-surgery. Control, HDs.

To mitigate the influence of contaminating cells, 7.5 mL of PB from HDs (*n* = 5) was processed for CTC enrichment in the same way as ESCC samples (*n* = 77) and used as controls. All the target genes were expressed at lower levels in output cell-fraction in HDs, compared with those in ESCC patients (Figure 3A). For better visualization of the gene expression profiling data, a heatmap was generated by categorizing relative fold change values into three levels: negative (<2), positive (≥2), and high (≥5) (Figure 3B). *TWIST1*, *MET*, and *VEGFC* were the top three sensitive marker genes among the panels with high overall positivity in detecting CTCs in ESCC. For dynamic monitoring of the progression of disease (PD) in ESCC patients, 22 pairs of PB samples, including baseline and corresponding pre-III or one-month post-treatment samples, were clustered and presented in the heatmap (Figure 3C). In the palliative cohort (*n* = 9), a total of 56% (5/9) of patients exhibited a decrease in CTC markers at the pre-III time point. In the curative cohort (*n* = 13), this percentage was slightly higher, with nearly 70% (9/13) of patients showing decreased CTC markers at the one-month post-treatment time point. While the majority of samples displayed a reduction in the number of detected markers at the one-month post-treatment time point, four cases (C1b, C2b, C6b, and C12b) demonstrated an increase even after curative treatment. Notably, most of these cases experienced PD within 3 months after the later time point. The prognostic value of gene expression in CTCs at the post-treatment time point was further evaluated. The CTC-high status was found to be significantly correlated with PD in the curative cohort (1/8, 12.5% vs. 4/5, 80.0%, Fisher’s exact test, *p* = 0.032, two-sided). Furthermore, both *TWIST1*-positive (2/8, 25.0% vs. 5/5, 100.0%, Fisher’s exact test, *p* = 0.021, two-sided) and *TWIST1*-high (0/8, 12.5% vs. 3/5, 60.0%, Fisher’s exact test, *p* = 0.035, two-sided) statuses were found to be significantly correlated with PD in this cohort.

### 3.3. Correlations of CTC Marker Expression with Clinicopathological Features in ESCC Patients

The correlations between patients’ clinical characteristics and different groups of CTC marker expression are summarized in Table 1. A total of 55 ESCC patients were enrolled. The median progression-free survival (PFS) and overall survival (OS) among palliative patients were 84 days and 119 days, respectively. For curative patients, the median PFS and OS were 243 days and 299 days, respectively. Approximately 84% (46/55) of patients were CTC-positive and this status was significantly correlated with original tumor size (*p* = 0.031). Regarding the clinicopathological correlation with individual CTC markers, positive expression of *TWIST1*, *KRT5*, and *KRT18* showed significant correlations with advanced pathological stages (III-IV) (*p* < 0.001, *p* = 0.001, and *p* = 0.026, respectively). Positive expression of *MET* was significantly linked to larger tumor size (69% vs. 30%, *p* = 0.033). Furthermore, positive expression of *TWIST1*, *KRT5*, and *FSCN1* demonstrated significant correlations with both advanced node metastasis (79% vs. 41%, *p* = 0.006, 68% vs. 22%, *p* = 0.001, and 57% vs. 26%, *p* = 0.029) and distant metastasis (93% vs. 49%, *p* = 0.004, 86% vs. 32%, *p* < 0.001, and 79% vs. 29%, *p* = 0.02). However, no correlation was found between the expression of individual CTC markers and the age, gender, or original tumor location of ESCC patients.

### 3.4. Correlation of CTC Marker Expression at Baseline with PFS or OS in ESCC Patients

**Figure 4:**
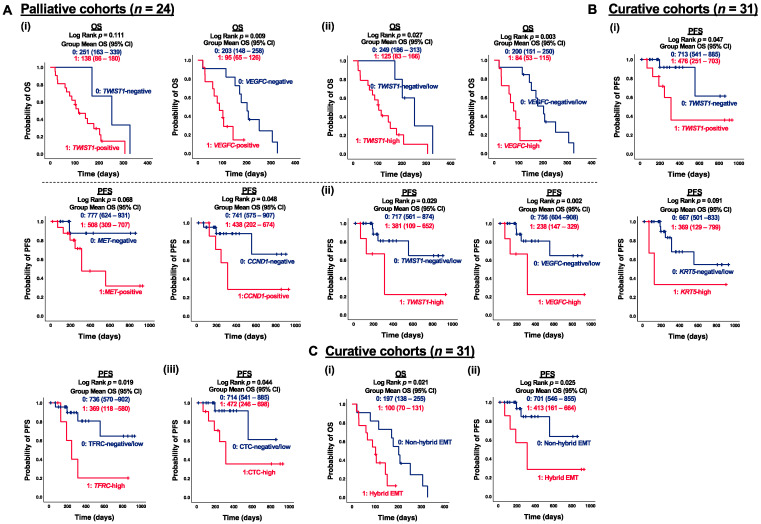
Correlation of CTC marker expression at baseline with PFS or OS in ESCC subgroups. (**A**) Kaplan–Meier survival analysis of OS with CTC gene expression at baseline in palliative cohorts (*n* = 24). Gene stratification into two groups, (**i**) 0, 1: negative and positive gene expression; (**ii**) 0, 1: negative/low and high gene expression. (**B**) Kaplan–Meier survival analysis of PFS with CTC gene expression at baseline in curative cohorts (*n* = 31). Gene stratification into two groups, (**i**) 0, 1: negative and positive gene expression; (**ii**) 0, 1: negative/low and high gene expression; (**iii**) 0, 1: CTC-negative/low and CTC-high. (**C**) (**i**) Kaplan–Meier survival analysis of PFS with hybrid EMT CTC at baseline in palliative cohorts (*n* = 24), (**ii**) Kaplan–Meier survival analysis of OS with hybrid EMT CTC at baseline in curative cohorts (*n* = 31). Gene stratification into two groups 0, 1: non-hybrid and hybrid EMT CTC.

**Figure 5:**
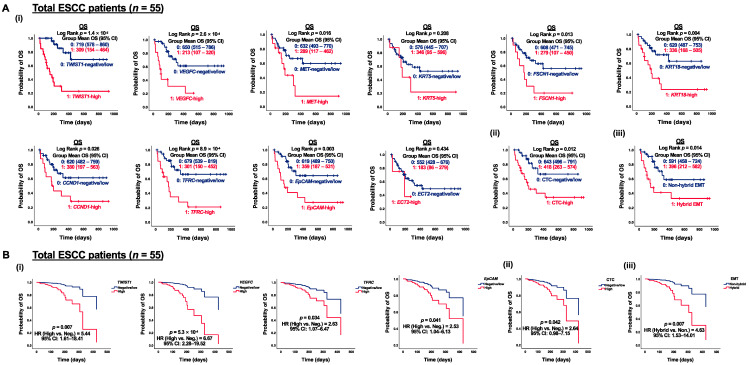
Correlation of CTC marker expression at baseline with OS in ESCC patients (*n* = 55). (**A**) Kaplan–Meier survival analysis of OS with CTC gene expression at baseline in ESCC patients (*n* = 55). Gene expression stratification into two groups, (**i**) 0, 1: negative/low and high gene expression; (**ii**) 0, 1: CTC-negative/low and CTC-high; (**iii**) 0, 1: non-hybrid and hybrid EMT CTC. (**B**) Cox regression survival analysis of OS with CTC gene expression at baseline in ESCC patients (*n* = 55). Gene expression stratification into two groups, (**i**) 0, 1: negative/low and high gene expression; (**ii**) 0, 1: CTC-negative/low and CTC-high; (**iii**) 0, 1: non-hybrid and hybrid EMT CTC.3.5. Functional Role of TWIST1 in ESCC Metastatic Progression.

Although limited correlations between CTC marker expression and clinicopathological features were observed within the palliative cohort (*n* = 24), the prognostic impact of CTC marker expression on OS can still provide insights into risk factors and essential molecular events during ESCC progression. Notably, both *TWIST1* and *VEGFC* were significant prognostic factors for OS in patients receiving palliative care. Specifically, positive expression of *VEGFC* was significantly correlated with shorter OS in palliative patients (*p* = 0.009) (Figure 4(Ai)). High expression of *TWIST1* and *VEGFC* were both significantly associated with shorter OS in palliative patients, with *p*-values of 0.027 and 0.003, respectively (Figure 4(Aii)). For dynamic treatment monitoring, predictive and prognostic markers to evaluate the treatment efficacy are still urgently needed. Among the curative cohorts (*n* = 31), patients with positive expression of *TWIST1* and *CCND1* exhibited significantly shorter PFS than those with negative detection (*p* = 0.047 and *p* = 0.048, respectively) (Figure 4(Bi)). In terms of individual gene analysis, high expression of CTC markers, defined as fold changes greater than five, demonstrated a stronger correlation with PFS. Specifically, patients with high expression of *TWIST1*, *VEGFC*, and *TFRC* had significantly shorter PFS compared to those with negative/low expression (*p* = 0.029, *p* = 0.002, and *p* = 0.019, respectively) (Figure 4(Bii)). Furthermore, patients with at least five detected CTC markers (CTC-high) exhibited significantly shorter PFS compared to non-CTC-high patients (*p* = 0.044) (Figure 4(Biii)). Patients with CTCs co-expressing *TWIST1* and *EpCAM* were classified as hybrid EMT. Patients with hybrid EMT CTC phenotype had significantly shorter OS (*p* = 0.021) and PFS (*p* = 0.025) in the palliative and curative cohorts, respectively, as compared to the opposites (Figure 4C). Both *TWIST1* and *VEGFC* served as prognostic factors for PFS in curative patients and OS in palliative patients. To further illustrate the clinical significance of CTC markers in ESCC, a univariate Kaplan–Meier survival analysis of OS with CTC gene expression at baseline was conducted prior to multivariate Cox regression analysis. CTC gene expression was stratified into two groups, with 0 indicating negative/low and 1 indicating high gene expression. Patients in the group with high expression of *TWIST1*, *VEGFC*, *MET*, *FSCN1*, *KRT18*, *CCND1*, *TFRC*, and *EpCAM* at baseline exhibited significantly shorter OS than the opposite group (Figure 5(Ai)). Patients classified as CTC-high had a markedly shorter OS, compared to the CTC-negative/low group (418 days vs. 643 days, *p* = 0.012) (Figure 5(Aii)). Similarly, patients grouped into hybrid EMT showed a significantly shorter OS than the non-hybrid group (396 days vs. 591 days, *p* = 0.014) (Figure 5(Aiii)). Clinicopathological features, including gender, age, TNM stages, and treatment classification, were included as covariates in the CTC multivariate Cox regression models for OS analysis. Multivariate regression model analysis indicated that high expression of *TWIST1* (Hazard ratio (HR) for *TWIST1*-high 5.44, *p* = 0.007), *VEGFC* (HR for *VEGFC*-high 6.67, *p* < 0.001), *TFRC* (HR for *TFRC*-high 2.63, *p* = 0.034), and *EpCAM* (HR for *EpCAM*-high 2.53, *p* = 0.041) at baseline were significantly associated with a higher OS (Figure 5(Bi)). Furthermore, CTC-high (HR for CTC-high 2.64, *p* = 0.042) (Figure 5B(ii)) and hybrid EMT (HR for hybrid EMT 4.63, *p* = 0.007) were identified as unfavorable independent prognosticators in terms of OS in ESCC (Figure 5(Biii)).

**Figure 6:**
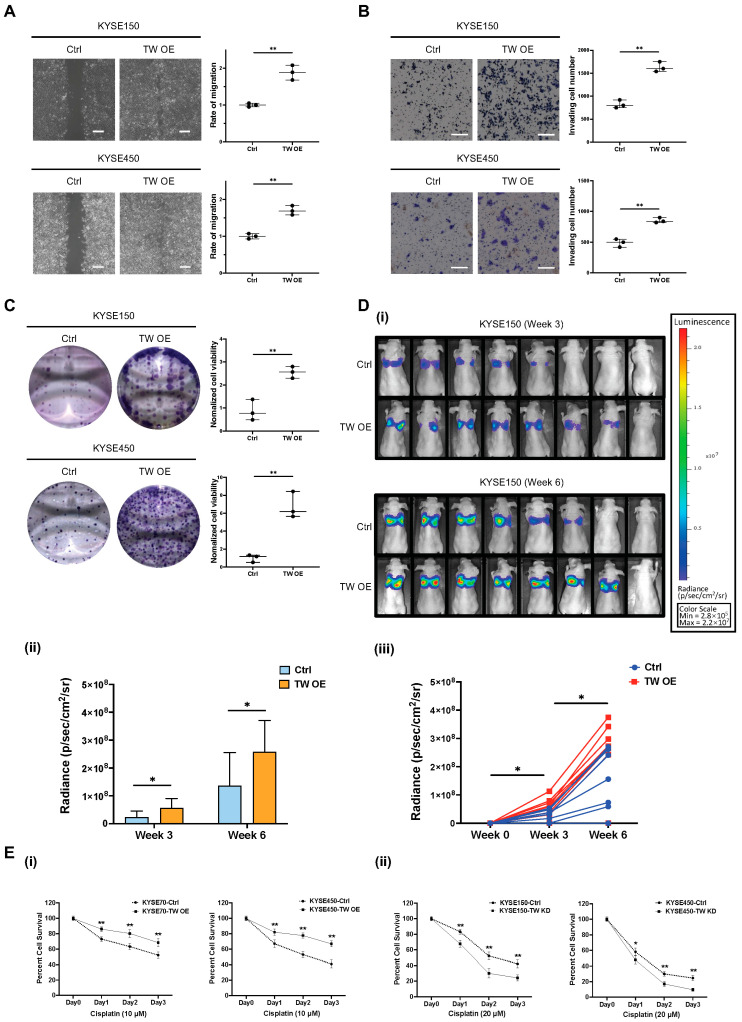
Functional role of *TWIST1* in ESCC metastatic progression. (**A**) A wound healing assay was performed to determine the cell migration ability of *TWIST1*-overexpressing cells. Magnification: 4×. Scale bars: 200 μm. Data show means ± SD of three independent experiments. Ctrl: control; TW OE: *TWIST1* overexpression. ** *p*-value < 0.01. (**B**) A Matrigel-coated transwell assay was performed to determine the cell invasion ability of *TWIST1*-overexpressing cells. Magnification: 10×. Scale bars: 200 μm. Data show means ± SD of three independent experiments. ** *p*-value < 0.01. (**C**) A colony formation assay was performed to determine the cell clonogenicity of *TWIST1*-overexpressing cells. Data show means ± SD of three independent experiments. ** *p*-value < 0.01. (**D**) Bioluminescence signals were used to evaluate the in vivo metastasis of *TWIST1*-overexpressing cells. (**i**) All images were taken under 1 min exposure and adjusted to the same scale. (**ii**) Metastasis quantification. * *p*-value < 0.05. (**iii**) Speed of metastasis of *TWIST1*-overexpressing cells. * *p*-value < 0.05. (**E**) An MTT assay was performed to determine the cell viability of cells treated with (**i**) 20 μM cisplatin and (**ii**) 10 μM cisplatin for 24, 48, 72 h. Data show the means ± SD of three independent experiments. TW KD: *TWIST1* knockdown. ** *p*-value < 0.01, * *p*-value < 0.05.

*TWIST1* was identified as one of the most sensitive marker genes for characterizing mesenchymal CTCs in ESCC, as described in this study. The functional role of *TWIST1* in ESCC progression and development was further investigated both in vitro and in vivo using CRISPR systems. To confirm the involvement of EMT progression of *TWIST1* in ESCC, we first overexpressed *TWIST1* in KYSE150 and KYSE450 cells (Figure S3). The overexpression of *TWIST1* significantly increased the cell migration ability, as indicated by the wound healing assay. Compared to the control, the migration rate increased to 189% for KYSE150 cells and 168% for KYSE450 cells upon the *TWIST1* overexpression (Figure 6A). The invasion assay revealed that overexpression of *TWIST1* drastically increased the ability of cell invasion in ESCC. The relative invasive ability increased to 198% for KYSE150 cells and 174% for KYSE450 cells upon *TWIST1* overexpression, as compared with the control (Figure 6B). Furthermore, the clonogenicity ability was significantly enhanced in both KYSE150 cells and KYSE450 cells upon *TWIST1* overexpression, as assessed by the colony formation assay (Figure 6C). Additionally, more metastatic formations were detected in Luc-KYSE 150 cells upon *TWIST1* overexpression. Higher bioluminescence signal intensity was observed in the *TWIST1* overexpression group at both weeks 3 and 6 post-injection, highlighting the in vivo pro-metastatic role of *TWIST1* in ESCC (Figure 6D). In addition to the enhanced ability of cell migration, invasion, and clonogenicity, *TWIST1*-induced chemo-resistance was further investigated in ESCC. *TWIST1*-knockdown KYSE150 and KYSE450 cells were incubated with varying concentrations of cisplatin, 5-FU, paclitaxel, and docetaxel for 48 h. Notably, *TWIST1* was found to be specifically linked to chemo-resistance towards cisplatin in ESCC. Knockdown of *TWIST1* significantly sensitized cells to cisplatin treatment (Figure 6(Ei)), while overexpression of *TWIST1* significantly escalated the resistance of ESCC cells to cisplatin (Figure 6(Eii)). The differential expression between the overexpression and knockdown groups for *TWIST1* in ESCC cell lines is shown in Table S8.

### 3.5. Functional Role of TWIST1-TGFBI-ZEB1 Signaling in ESCC

**Figure 7:**
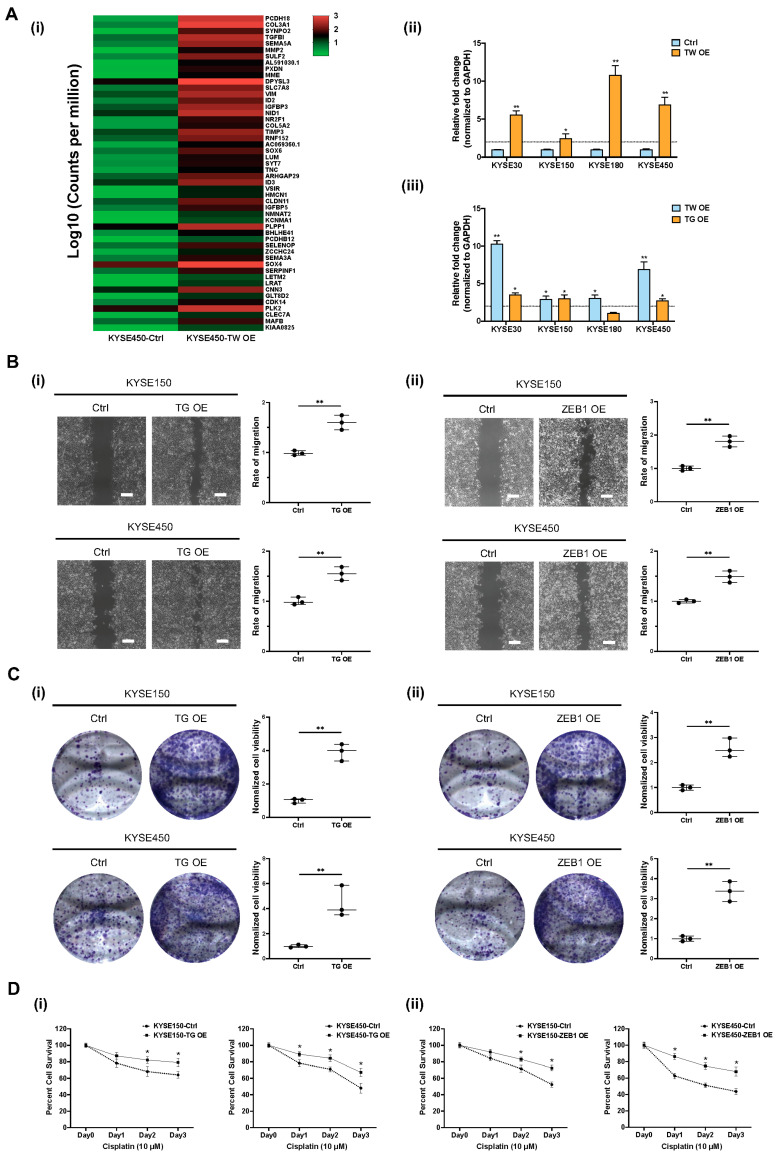
Functional role of *TWIST1-TGFBI-ZEB1* signaling in ESCC. (**A**) *TGFBI-ZEB1* is regulated by *TWIST1* in ESCC, as indicated by RNA-seq and RT-qPCR. (**i**) RNA-seq heat map of top 50 upregulated genes upon *TWIST1* overexpression in KYSE450 cells. (**ii**) Transcript level of *TGFBI* downstream of *TWIST1* was quantified using RT-qPCR. Error bars represent standard error mean. ** *p*-value < 0.01, * *p*-value < 0.05. (**iii**) overexpression. ** *p*-value < 0.01, * *p*-value < 0.05. (**B**) Wound healing assay was performed to determine the cell migration ability of (**i**) *TGFBI*-overexpressing cells and (**ii**) *ZEB1*-overexpressing cells. Magnification: 4×. Scale bars: 200 μm. Data show means ± SD of three independent experiments. ** *p*-value < 0.01. (**C**) Colony formation assay was performed to determine the cell clonogenicity of (i) *TGFBI*-overexpressing cells and (ii) *ZEB1*-overexpressing cells. Data show means ± SD of three independent experiments. ** *p*-value < 0.01. (**D**) MTT assay was performed to determine cell viability of (i) *TGFBI*-overexpressing cells and (ii) *ZEB1*-overexpressing cells treated with 10 μM cisplatin for 24, 48, 72 h. Data show the means ± SD of three independent experiments. * *p*-value < 0.05.

To identify downstream signaling pathways regulated by *TWIST1*, ribo-depleted RNA-sequencing was performed in KYSE450 cells. Based on the transcriptomic analysis, a heatmap of the top 50 upregulated genes was generated according to fold changes upon *TWIST1* overexpression (Figure 7(Ai)). The upregulated genes were further subjected to EnrichR pathway analysis and *TGFBI* was highlighted as one of the most enriched downstream target genes upon *TWIST1* overexpression (Figure S4). Interestingly, the *TGFBI-ZEB1* axis was previously reported to protect against genetic stress in breast cancer [21]. Therefore, the regulatory relationship between *TWIST1* and the *TGFBI-ZEB1* axis was further investigated in ESCC. The level of *TGFBI* mRNA was significantly upregulated upon *TWIST1* overexpression in KYSE30, KYSE150, KYSE180, and KYSE450 cells, as verified by the RT-qPCR (Figure 7(Aii)). The overexpression of either *TWIST1* or *TGFBI* significantly increases the transcript level of *ZEB1* in various ESCC cell lines (Figure 7(Aiii)). The functional roles of *TGFBI* and *ZEB1* downstream of *TWIST1* were studied using lentiviral-mediated CRISPR activation in KYSE150 and KYSE450 cells (Figures S5 and S6). Significant increases in cell migration ability were observed both in *TGFBI*-overexpressing cells and *ZEB1*-overexpressing cells when compared with the corresponding controls, as indicated by the wound healing assay (Figure 7B). Overexpression of *TGFBI* and *ZEB1* also drastically enhanced the cell clonogenicity, as indicated by the colony formation assay both in KYSE150 cells and KYSE450 cells (Figure 7C). *TWIST1* conferred chemo-resistance to cisplatin treatment in ESCC. We further investigated whether *TGFBI*-*ZEB1* was involved in the *TWIST1*-induced cisplatin resistance. Cell viability of *TGFBI*-overexpressing cells and *ZEB1*-overexpressing cells incubated with cisplatin was determined using the MTT assay (Figure 7D). Compared to the corresponding controls, overexpression of both *TGFBI* and *ZEB1* induced cisplatin resistance in ESCC, and a significant difference in cell viability was observed, when incubated with 10 μM cisplatin for 48 h or longer.

## 4. Discussion

We evaluated a novel identified panel of 10-gene transcripts present in CTCs of ESCC patients to investigate its clinical significance. As in our previous study, antibodies for Cytokeratin, EpCAM, MUC1, and VIM proteins were used for immunofluorescence enumeration of CTCs in ESCC and confirmed that elevated CTC levels at both pre- and post-treatment stages showed significant and independent prognostic value during the longitudinal monitoring of CTC enumeration [10]. In the present study, *CCND1*, *ECT2*, *EpCAM*, *FSCN1*, *KRT5*, *KRT18*, *MET*, *TFRC*, *TWIST1*, and *VEGFC* were identified as CTC biomarkers based on NGS bioinformatic analysis, spike-in assays and clinical validation in ESCC CTCs considering sensitivity and specificity. Typically, transcripts of the novel 10-gene panel, with the exception of ECT2, correlate with clinicopathological parameters or patient survival, when present in CTCs enriched from PB samples. The low detection rate and insignificant clinical relevance of *ECT2* might be attributed to the discrepancy of endogenous gene expression between ESCC cell lines and clinical CTCs. Compared to the EpCAM-dependent CellSearch^®^ system, the label-free, size-dependent CTC isolation method was found superior in sensitivity for downstream molecular characterization of CTCs in head and neck squamous cell carcinoma (HNSCC) [22]. Centrifugal force in CTChip^®^FR1 microfluidic chips were used to enrich and separate larger CTCs based on size from PBMCs in 77 PB samples from 55 ESCC patients in this study, resulting in an overall detection rate of 81.8% (63/77) for CTC-positive samples and 50.6% (39/77) for CTC-high samples. Among the 39 CTC-high samples, 59% (23/39) were from palliative patients, while 41% (16/39) were from curative patients. The lower occurrence of CTC-high samples among the curative patients, when compared to palliative patients, can be attributed to insufficient or even absence of CTCs, making their isolation challenging.

Previous studies on ESCC have employed molecular assays to detect CTCs by examining mRNA levels through RT-qPCR, yielding positive detection rates ranging from 14% to 60% [23,24,25,26]. Variations in these detection rates could be attributed to differences in CTC isolation techniques, the characteristics of the isolated CTC population based on the method used, and the CTC markers utilized for characterization. For instance, in spike-in assays, epithelial markers *KRT18*, *KRT19*, *MET*, and *MUC1* demonstrated higher sensitivity in KYSE30 cells, while the EMT regulators *SNAI2* and *TWIST1* exhibited greater sensitivity in KYSE270 cells due to the distinct EMT status between these cell lines (Figure 2B). Besides CTCs that express epithelial antigens, a subset of CTCs possesses an EMT phenotype, which might have existed, but remained undetectable when using detection methods relying exclusively on epithelial markers. EMT markers are particularly prevalent in highly heterogeneous CTCs, which can only be captured using unbiased enrichment strategies. Therefore, focusing on CTC markers associated with EMT could offer novel insights for improved identification and characterization. The overall detection rate of EMT markers is higher than that of epithelial markers, standing at 43.8% (46/105) versus 29.5% (31/105), as evidenced by multiplex gene expression profiling of CTCs in high-risk prostate cancer [27].

Various studies have affirmed the prognostic significance of mesenchymal CTCs for distant metastasis and survival across multiple cancers, including HNSCC, pancreatic ductal adenocarcinoma (PDAC), and breast cancer [22,28,29,30]. In PDAC, the presence of mesenchymal-CTCs, characterized by *VIM* and *TWIST1*, was positively correlated with the TNM stage (*p* < 0.01) and distant metastasis (*p* < 0.01) [28]. Additionally, the prevalence of mesenchymal markers, including *TWIST1* and *VIM*, was also underscored in breast cancer. Among the 84.9% (920/1083) of cases positive for CTCs, 547 displayed epithelial CTCs, 793 showed hybrid EMT CTCs, and 516 exhibited mesenchymal CTCs, highlighting how EMT imparts a more invasive and metastatic phenotype to CTCs [30]. Furthermore, both biphenotypic and mesenchymal CTCs were associated with distant metastasis, as indicated by ROC curve analysis. Similarly, the results in the present study showed the overall mRNA detection rate of the EMT marker *TWIST1* was higher than that of the epithelial marker *EpCAM*: 62.3% (48/77) vs. 41.6% (32/77). High expression of *TWIST1* was detected in nearly half of the patients (26/55) and was more frequently observed in patients at advanced ESCC stages (67% vs. 18%, *p* < 0.001), as well as in patients with distant metastasis (86% vs. 34%, *p* = 0.001). The high expression of *TWIST1* in CTCs was significantly correlated with shorter OS (*p* = 0.027) in palliative patients and shorter PFS (*p* = 0.029) in curative patients.

Characterized as hybrid EMT or biphenotypic, this intermediate state between the epithelial and mesenchymal phenotypes exhibits the highest metastatic potential [31,32]. In ESCC, biphenotypic and mesenchymal CTCs are dominant subtypes, with epithelial markers (*EpCAM* and *CK8/18/19*) and mesenchymal markers (*TWIST1* and *VIM*) identifying 58.9% (76/129) biphenotypic CTCs and 32.6% (42/129) mesenchymal CTCs [33]. Our data found 36.4% (20/55) of ESCC patients detected with hybrid EMT CTC at baseline. Importantly, hybrid EMT CTC significantly correlates with shorter OS in palliative patients and shorter PFS in curative patients in the current study. Our CTC multivariate Cox regression models for OS analysis also revealed the prognostic value of hybrid EMT at baseline (HR for Hybrid EMT 4.63, *p* = 0.007). *VEGFC* is another CTC marker highlighted in this study for its high sensitivity and significant prognostic value. By activating its receptor VEGFR3 expressed on lymphatic endothelial cells, *VEGFC* plays a key role in promoting lymphangiogenesis and subsequent regional lymph node metastasis in various human cancers, including ESCC [34]. Our data found both positive expression of *VEGFC* (*p* = 0.009) and high expression of *VEGFC* (*p* = 0.003) in CTCs were significantly associated with shorter OS in palliative patients. High expression of *VEGFC* was also an independent prognosticator in terms of OS (HR for *VEGFC*-high 6.67, *p* < 0.001), as indicated in CTC multivariate Cox regression models.

In line with prognostic markers observed in clinical PB samples, *TWIST1*, *VEGFC*, and *CCND1* demonstrated high sensitivity in characterizing CTCs at the transcript level in cardiac blood from PDOX models. These gene expression profiling data in CTCs reinforced the importance of the EMT regulator *TWIST1* and the lymphatic metastatic promotor *VEGFC* in ESCC. Furthermore, the involvement of the *CCND1* gene in facilitating cell motility should also be fortified, as previously reported in breast cancer [35].

Distinct morphological, transcriptional, and epigenetic features occur along with EMT progression. Chemoresistance, one of the major obstacles during clinical cancer therapy, can be triggered by EMT, as indicated by increasing molecular and phenotypic evidence [36,37,38]. Including cisplatin and paclitaxel, *TWIST1* has also been linked to resistance to platinum-based chemotherapy in several cancer types [37,39,40]. Additionally, the knockdown of *TWIST1* has been shown to partially reverse multidrug resistance in breast cancer cells [36]. In the current study, the relationship between *TWIST1* and drug resistance to conventional chemotherapy, including cisplatin, 5-FU, paclitaxel, and docetaxel was further investigated. Lentiviral-mediated CRISPR activation and interference were used for direct gene editing of *TWIST1*, revealing its influence on treatment efficacy in ESCC was observed mainly with cisplatin. Specifically, *TWIST1* depletion sensitized cells to cisplatin treatment, whereas *TWIST1* overexpression induced cisplatin drug resistance in ESCC. Similar evidence was also observed in epithelial ovarian cancer, where among more mesenchymal differentiated cells, *TWIST1* might actually lead to greater resistance to cisplatin [38]. These collective discoveries establish a connection between *TWIST1* and the EMT process, demonstrating their dual malignant roles in promoting heightened metastasis and drug resistance, underscoring the attractiveness of *TWIST1* as a particularly compelling target for combination therapeutic approaches in aggressive and drug-resistant carcinomas.

To elucidate the underlying molecular mechanism of *TWIST1*-initiated EMT progression and cisplatin resistance in ESCC, ribo-depleted RNA-sequencing, and EnrichR pathway analysis were conducted. Our bioinformatic analysis of transcriptomic data highlighted *TGFBI* as one of the most enriched downstream targets of *TWIST1*, which was further validated by RT-qPCR in four ESCC cell lines. Importantly, *TGFBI* is one of the direct transcriptional targets of *TWIST1*, as validated by both ChIP-PCR using mouse embryonic tissues and luciferase assays [41]. Playing a role in preserving mesenchymal cell traits through intracellular signaling and extracellular matrix (ECM) remodeling, *TGFBI* influences cell adhesion and migration capabilities in a range of cancers, including intestinal cancers, glioma, and melanoma [42]. Necessary for tumor sphere formation and chromosomal stability maintenance, *TGFBI*-*ZEB1* was reported to protect against genetic stress of activated stem-like cells in breast cancer [21]; their involvement downstream of *TWIST1* in ESCC was investigated. As indicated by qPCR, both mRNA levels of *TGFBI* and *ZEB1* were upregulated upon *TWIST1* overexpression, and overexpression of *TGFBI* significantly increased the transcript level of *ZEB1* in ESCC. Gain-of-function studies of *TGFBI* and *ZEB1* in ESCC highlighted their potential for increasing the cell migration and clonogenicity in ESCC cells upon overexpression, as indicated by wound healing and colony formation assays. Moreover, *TGFBI*-*ZEB1* was involved in the *TWIST1*-induced cisplatin resistance. Overexpression of either *TGFBI* or *ZEB1* conferred cisplatin resistance to ESCC cells, including KYSE150 and KYSE450 cells.

We now demonstrate that *TWIST1* mRNA expression in CTCs is a significant prognostic marker for patient survival, and meanwhile, *TWIST1* promotes EMT and confers cisplatin resistance through the *TWIST1-TGFBI-ZEB1* signaling pathway in ESCC. Although *TWIST1* itself is not directly targetable, targeting the effector proteins of this alternative signaling cascade, such as TGFBI, may offer a promising therapeutic strategy. *TGFBI* has the potential to be a therapeutic target and could partially explain its clinical benefit in preventing metastatic recurrence and cisplatin resistance. In ovarian cancer, treatment with anti-TGFBI antibodies has been demonstrated to reduce peritoneal tumor size in an orthotopic mouse model [43]. Radiolabeled anti-TGFBI antibodies have demonstrated anti-angiogenic activity in colorectal cancer by selectively targeting metastatic lesions in vivo, highlighting its diagnostic and therapeutic potential [44]. Our study confirms that *TGFBI* is a therapeutic target downstream of *TWIST1* in ESCC, and, importantly, that *TGFBI* overexpression confers cisplatin resistance in ESCC. Targeting *TGFBI* in advanced or metastatic ESCC has the potential to simultaneously inhibit metastatic potential and cisplatin resistance, thereby reversing cancer progression and improving the treatment efficacy.

A limitation of this study was the small number of healthy controls and the inadequate follow-up of CTCs for longitudinal analysis. Therefore, future studies with a larger cohort and healthy control groups, as well as a longer-term follow-up will strengthen current findings on the efficacy of these markers. Another limitation was the possible underestimation of total CTCs since the size-based capture platform utilized is biased toward the recovery of larger CTCs. The size-based capture platform is incapable of distinguishing small CTCs that are comparable in size with PBMCs, thus leading to possible underestimation of the total number of CTCs.

## 5. Conclusions

The present study identified a 10-gene panel for CTC identification in ESCC, namely *CCND1*, *ECT2*, *EpCAM*, *FSCN1*, *KRT5*, *KRT18*, *MET*, *TFRC*, *TWIST1*, and *VEGFC*. Expression of these CTC markers significantly correlates with clinicopathological features and patient survival. Molecular studies showed that *TWIST1*-initiated EMT progression and cisplatin resistance can partially be explained by *TWIST1-TGFBI-ZEB1* signaling. Additionally, targeting the downstream effector protein TGFBI emerges as an ideal strategy to simultaneously inhibit metastatic potential and reverse cisplatin resistance. These findings collectively provide valuable insights into the understanding of ESCC progression and benefit patients with improved interventions based on these therapeutic targets.

## Figures and Tables

**Table 1:** Correlations of CTC marker expression with clinicopathological features in patients with ESCC.

Clinical Parameters	Patients (*n* = 55)	CTC-Positive ^b^	*TWIST1* ^c^	*MET*	*VEGFC*	*KRT5*	*FSCN1*	*KRT18*	*CCND1*	*TFRC*	*EpCAM*	*ECT2*
	(within group %)	46 (83.6)	33 (60.0)	34 (61.8)	25 (45.5)	25 (45.5)	23 (41.8)	33 (60.0)	22 (40.0)	29 (52.7)	25 (45.5)	14 (25.5)
Median age (range)	68 ± (33–82)											
≤68	28 (50.9)	24 (85.7)	19 (67.9)	19(67.9)	13 (46.4)	16 (57.1)	11 (39.3)	19 (67.9)	15 (53.6)	14 (50.0)	12 (42.9	7 (25.0)
>68	27 (49.1)	22 (81.5)	14 (51.9)	15 (55.6)	12 (44.4)	9 (33.3)	12 (44.4)	14 (51.9)	7 (25.9)	15 (55.6)	13 (48.1)	7 (25.9)
Gender												
Female	6 (10.9)	5 (83.3)	5 (83.3)	4 (66.7)	2 (33.3)	4 (66.7)	3 (50.0)	5 (83.3)	2 (33.3)	3 (50.0)	1 (16.7)	1 (16.7)
Male	49 (89.1)	41 (83.7)	28 (57.1)	30 (61.2)	23 (46.9)	21 (42.9)	20 (40.8)	28 (57.1)	20 (40.8)	26 (53.3)	24 (49.0)	13 (26.5)
Tumor location												
Lower	25 (45.5)	19 (76.0)	15 (60.0)	15 (60.0)	10 (40.0)	13 (52.0)	15 (40.0)	16 (64.0)	8 (32.0)	11 (44.0)	10 (40.0)	4 (16.0)
Upper/Middle	30 (54.5)	27 (90.0)	18 (60.0)	19 (63.3)	15 (50.0)	12 (40.0)	13 (43.3)	17 (56.7)	14 (46.7)	18 (60.0)	15 (50.0)	10 (33.3)
TNM stage **^a^**			***p* ^d^ < 0.001**			***p* = 0.001**		***p* = 0.026**				
0–II	22 (40.0)	17 (81.0)	6 (27.3)	10 (45.5)	9 (40.9)	4 (18.2)	6 (27.3)	9 (40.9)	6 (27.3)	11 (50.0)	9 (40.9)	5 (22.7)
III–IV	33 (60.0)	29 (85.3)	27 (81.8)	24 (72.7)	16 (48.5)	21 (63.6)	17 (51.5)	24 (72.7)	16 (48.5)	18 (54.5)	16 (48.5)	9 (27.3)
Tumor size		***p* = 0.031**		***p* = 0.033**								
T0–T2	10 (18.2)	5 (55.6)	3 (30.0)	3 (30.0)	2 (20.0)	4 (40.0)	3 (30.0)	5 (50.0)	2 (20.0)	3 (30.0)	2 (20.0)	0 (0.00)
T3–T4	45 (81.8)	41 (89.1)	30 (66.7)	31 (68.9)	23 (51.1)	21 (46.7)	20 (44.4)	28 (62.2)	20 (44.4)	26 (57.8)	23 (51.1)	14 (31.1)
Node stage			***p* = 0.006**			***p* = 0.001**	***p* = 0.029**					
N0–N1	27 (49.1)	22 (84.6)	11 (40.7)	13 (48.1)	11 (40.7)	6 (22.2)	7 (25.9)	14 (51.9)	9 (33.3)	13 (48.1)	13 (48.1)	5 (18.5)
N2–N3	28 (50.9)	24 (82.8)	22 (78.6)	21 (75.0)	14 (50.0)	19 (67.9)	16 (57.1)	19 (67.9)	13 (46.4)	16 (57.1)	12 (42.9)	19 (32.1)
Distant Metastasis			***p* = 0.004**			***p* < 0.001**	***p* = 0.002**	***p* = 0.029**				
NO	41 (74.5)	32 (78.0)	20 (48.8)	22 (53.7)	18 (43.9)	13 (31.7)	12 (29.3)	21 (51.2)	13 (31.7)	19 (46.3)	16 (39.0)	8 (19.5)
YES	14 (25.5)	14 (100.0)	13 (92.9)	12 (85.7)	7 (50.0)	12 (85.7)	11 (78.6)	12 (85.7)	9 (64.3)	10 (71.4)	9 (64.3)	6 (42.9)

^a^ 8th edition of the American Joint Committee on Cancer (AJCC) TNM system for Cancer Staging. ^b^ CTC-positive, samples with at least 2 detected markers. ^c^ Fold change ≥ 2.0 with the 2^−ΔΔCt^ method. ^d^ Fisher exact test, 2-sided. Bold if *p*-value < 0.05.

## Data Availability

The data presented in this study are available on request from the corresponding author.

## Supplementary Materials

The following supporting information can be downloaded at: https://www.mdpi.com/article/10.3390/cancers15225329/s1, Table S1: List of the primers used for RT-qPCR; Table S2: List of the primers used for gene cloning of *TWIST1*, *TGFBI*, and *ZEB1*; Table S3: List of sgRNA oligos designed for *TWIST1* knockdown using Lentiviral-mediated CRISPR interference; Table S4: List of the antibodies used in the study; Table S5: ROC analysis of CTC-positive and ESCC; Table S6: ROC analysis of CTC-high and survival; Table S7: Average reads of putative CTC markers in KYSE270 cells and PBMCs from 10× NGS sequencing; Table S8: Precise expression of *TWIST1* between the overexpression and knockdown groups in ESCC cell lines. Figure S1: Gene expression heatmap of 40 putative CTC markers by 10× NGS sequencing; Figure S2: Scores of Generic EMT Signature of 25 esophageal carcinoma cell lines; Figure S3: Validation of Lentiviral-mediated CRISPR activation and interference of *TWIST1* by Western blotting; Figure S4: EnrichR pathway analysis of top 100 *TWIST1*-upregulated genes in KYSE450 cells; Figure S5: Validation of Lentiviral-mediated CRISPR activation of *TGFBI* by RT-qPCR; Figure S6: Validation of Lentiviral-mediated CRISPR activation of *ZEB1* by RT-qPCR; Figures S7 and S8: Uncropped Western blot figures for Figure S3.
