# Supplementary material for: Multigene Profiling of Circulating Tumor Cells in Esophageal Squamous Cell Carcinoma Identifies Prognostic Cancer Driver Genes Associated with Epithelial-Mesenchymal-Transition Progression and Chemoresistance
Source: Cancers (Basel). 2023 Nov 8;15(22):5329. doi: 10.3390/cancers15225329 (PMC10670643; doi:10.3390/cancers15225329)
Supplement: Supplementary file 1 [file cancers-15-05329-s001.zip › cancers-2649494-supplementary.pdf]

List of Supplementary Tables and Figures

Supplementary Table S1: List of the primers used for RT-qPCR

Supplementary Table S2: List of the primers used for gene cloning of *TWIST1*, *TGFBI* and *ZEB1*

Supplementary Table S3: List of sgRNA oligos designed for *TWIST1* knockdown using Lentiviral-mediated CRISPR interference

Supplementary Table S4: List of the antibodies used in the study

Supplementary Table S5: ROC analysis of CTC-positive and ESCC

Supplementary Table S6: ROC analysis of CTC-high and survival

Supplementary Table S7: Average reads of putative CTC markers in KYSE270 cells and PBMCs from 10x NGS sequencing

Supplementary Table S8: Precise expression of *TWIST1* between the overexpression and knockdown groups in ESCC cell lines

Supplementary Figure S1: Gene expression heatmap of 40 putative CTC markers by 10x NGS sequencing

Supplementary Figure S2: Scores of Generic EMT Signature of 25 esophageal carcinoma cell lines

Supplementary Figure S3: Validation of Lentiviral-mediated CRISPR activation and interference of *TWIST1* by Western blotting

Supplementary Figure S4: EnrichR pathway analysis of top 100 *TWIST1*-upregulated genes in KYSE450 cells

Supplementary Figure S5: Validation of Lentiviral-mediated CRISPR activation of *TGFBI* by RT-qPCR

Supplementary Figure S6: Validation of Lentiviral-mediated CRISPR activation of *ZEB1* by RT-qPCR

Supplementary Figure S7–S8: Uncropped Western blot figures for Figure S3

Table S1: List of the primers used for qRT-PCR

Gene	Forward	Reverse	Extension temperature
GAPDH	GGTCTCCTCTGACTTCAACA	GTGAGGGTCTCTCTCTTCCT	60°C
GAPDH(mus)	CATCACTGCCACCCAGAAGACTG	ATGCCAGTGAGCTTCCCGTTCAG	60°C
PTPRC	CATATGACTATAACAGAGTGCC	ATGTATTTGCTTGGTTCCTC	60°C
PTPRC(mus)	CTTCAGTGGTCCCATTGTGGTG	TCAGACACCTCTGTCGCCTTAG	60°C
BAP1	GAGGATGACGTGCAGAACACCA	CTCAGCCAAGACGTTGATGGTG	60°C
BMP6	CCGACAACAGAGTCGTAATCGC	CTGCCATCCCAGGTCTTGGAAA	60°C
CCNE1	TGTGTCCTGGATGTTGACTGCC	CTCTATGTCGCACCACTGATACC	60°C
CDH1	TCACCACTGGGCTGGACCGA	TACAGCCTCCCACGCTGGGG	60°C
CDH2	ACAGTGGCCACCTACAAAGG	CCGAGATGGGGTTGATAATG	60°C
CTNNB1	CACAAGCAGAGTGCTGAAGGTG	GATTCCTGAGAGTCCAAAGACAG	60°C
CCND1	TCTACACCGACAACTCCATCCG	TCTGGCATTTTGGAGAGGAAGTG	60°C
DKK3	GTGCATCATCGACGAGGACTGT	TGGTCTCCACAGCACTCACTGT	60°C
ECT2	GCAGTCAGCAAGGTGGCAAGTT	CTCTGGTGCAAGGATAGGTCCA	60°C
EGFR	AACACCCTGGTCTGGAAGTACG	TCGTTGGACAGCCTTCAAGACC	60°C
EpCAM	AATCGTCAATGCCAGTGTACTT	TCTCATCGCAGTCAGGATCATAA	60°C
erbB2	GGAAGTACACGATGCGGAGACT	ACCTTCCTCAGCTCCGTCTCTT	60°C
FAT1	CAAACTCCCCTTCTGACAGC	CAGGGATCAAGATCCACCAC	60°C
FN1	GAAGCCGAGGTTTTAACTGC	ACCCACTCGGTAAGTGTTCC	60°C
FSCN1	GACACCAAAAAGTGTGCCTTCCG	CAAACTTGCCATTGGACGCCCT	60°C
FOXA1	GCAATACTCGCCTTACGGCTCT	GGGTCTGGAATACACACCTTGG	60°C
GATA4	GCGGTGCTTCCAGCAACTCCA	GACATCGCACTGACTGAGAACG	60°C
IL12B	GACATTCTGCGTTCAGGTCCAG	CATTTTTGCGGCAGATGACCGTG	60°C
ITGA6	CGAAACCAAGGTTCTGAGCCCA	CTTGGATCTCCACTGAGGCAGT	60°C
KCNH2	CATCTGCGTCATGCTCATTGGC	TCTGGTGGAAGCGGATGAACTC	60°C
KLF5	GGAGAAACGACGCATCCACTAC	GAACCTCCAGTCGCAGCCTTC	60°C
KRT8	ATCGAGATCACCACCTACCG	TGAAGCCAGGGCTAGTGAGT	60°C
KRT18	ACCCTCCAGACCTTGGAGAT	TCCATCTGTGCCTTGTATCG	60°C
KRT19	TGAGCAGGTCCGAGGTTACT	TCTTCCAAGGCAGCTTTCAT	60°C
LEF1	CTACCCATCCTCACTGTCAGTC	GGATGTTCCTGTTTGACCTGAGG	60°C
MET	TGCACAGTTGGTCCTGCCATGA	CAGCCATAGGACCGTATTTCGG	60°C
MMP3	CACTCACAGACCTGACTCGGTT	AAGCAGGATCACAGTTGGCTGG	60°C
MUC1	CCTACCATCCTATGAGCGAGTAC	GCTGGGTTTGTGTAAGAGAGGC	60°C
MYL9	GGATGTGATTCGCAACGCCTTTG	CGGTACATCTCGTCCACTTCCT	60°C
NETO2	GTAGTCGGCTTAGCAGGTTTCG	GGTATGCACAATTTTGGACACCAT	60°C
SERPINE1	CTCATCAGCCACTGGAAAGGCA	GACTCGTGAAGTCAGCCTGAAAC	60°C
SNAI1	TGCCCTCAAGATGCACATCCGA	GGGACAGGAGAAGGGCTTCTC	60°C

SNAI2	ATCTGCGGCAAGGCGTTTTCCA	GAGCCCTCAGATTTGACCTGTC	60℃
SOX2	CCCACCTACAGCATGTCCTACTC	TGGAGTGGGAGGAAGAGGTAAC	60℃
TFRC	ATCGGTTGGTGCCACTGAATGG	ACAACAGTGGGCTGGCAGAAAC	60℃
TGFB1	TACCTGAACCCGTGTTGCTCTC	GTTGCTGAGGTATCGCCAGGAA	60℃
TWIST1	GCCAGGTACATCGACTTCCTCT	TCCATCCTCCAGACCGAGAAGG	60℃
VEGFC	GCCAATCACACTTCCTGCCGAT	AGGTCTTGTTCGCTGCCTGACA	60℃
VIM	AGGCAAAGCAGGAGTCCACTGA	ATCTGGCGTTCCAGGGACTCAT	60℃
XPO1	CTACATCTGCCTCTCCGTTGCT	CCAATACTTCCTCTGGTTTAGCC	60℃
TGFBI	GGACATGCTCACTATCAACGGG	CTGTGGACACATCAGACTCTGC	60℃
ZEB1	GGCATACACCTACTCAACTACGG	TGGGCGGTGTAGAATCAGAGTC	60℃

Table S2: List of the primers used for gene cloning of *TWIST1*, *TGFBI* and *ZEB1*

Gene	**Forward primer sequence**	**Reverse primer sequence**
TWIST1	CGGGATCCGCCACCATGATGCAGGACGTGTCCAGCTCG	GCTCTAGACTAGTGGGACGCGGACATGGA
TGFBI	CGGAATTCGCCACCATGGCGCTCTTCGTGCGGCTGCTG	GCTCTAGACTAATGCTTCATCCTCTCTAA
ZEB1	CGGGATCCGCCACCATGAAAGTTACAAATTATAATACT	GCTCTAGATTAGGCTTCATTTGTCTTTTC

Table S3: List of sgRNA oligos designed for *TWIST1* knockdown using Lentiviral-mediated CRISPR interference

Name	Target sequence	Forward primer sequence	Reverse primer sequence
sgRNA1	CGGGAGTCCGCAGTCTTACG	CACCG CGGGAGTCCGCAGTCTTACG	AAAC CGTAAGACTGCGGACTCCCG C
sgRNA2	AGCGGGTCATGGCCAACGTG	CACCG AGCGGGTCATGGCCAACGTG	AAAC CACGTTGGCCATGACCCGCT C
sgRNA3	ACGAGGCGTTCGCCGCGCTG	CACCG ACGAGGCGTTCGCCGCGCTG	AAAC CAGCGCGGCGAACGCCTCGT C

Table S4: List of the antibodies used in the study

Antibody	Manufacturer	Catalog No.	Host	Application	Dilution factor
α-Tubulin	GeneTex	GTX112141	R	WB	1:10000
GAPDH	GeneTex	GTX100118	R	WB	1:10000
TWIST1	SANTA CRUZ	sc-81417	M	WB	1:1000

Table S5: ROC analysis of CTC-positive and ESCC

HD samples (n = 5)	CTC samples (n = 77)			
	AUROC	Threshold	Sensitivity	Specificity
	0.968	1.50	0.818	1

Table S6: ROC analysis of CTC-high and survival

Baseline (n = 55)	PFS				OS			
	AUROC	Threshold	Sensitivity	Specificity	AUROC	Threshold	Sensitivity	Specificity
	0.780	4.50	0.724	0.731	0.747	4.50	0.727	0.636

Table S7: Average reads of putative CTC markers in KYSE270 cells and PBMCs from 10x NGS sequencing

Gene	Average reads in KYSE270 cells	Average reads in PBMCs	*Gene*	Average reads in KYSE270 cells	Average reads in PBMCs
GAPDH	923.22	17.54	*MYL9*	6.30	0.02
ACTB	617.18	64.35	*CTNNA1*	6.14	0.22
RPLP0	363.02	21.50	*TPBG*	5.72	0.00
B2M	171.70	71.79	*IL18*	5.62	0.14
RPSA	146.00	15.90	*MSN*	5.58	1.66
NME2	123.14	3.31	*COL5A2*	5.54	0.00
PGK1	58.38	1.79	*STAT3*	5.50	0.43
RAC1	57.02	2.02	*MTA1*	5.46	0.12
KRT19	51.36	0.10	*IGFBP4*	5.40	0.05
KRT18	49.16	0.05	*TSPAN13*	5.18	0.15
KRT8	42.64	0.04	*GSK3B*	5.12	0.24
TIMP1	37.30	1.41	*PTP4A1*	4.94	0.16
CDKN2A	36.90	0.10	*BRMS1*	4.70	0.33
TNFSF10	33.38	0.72	*XPO1*	4.66	0.27
MYC	28.18	0.38	*CALD1*	4.66	0.00
SET	27.82	1.05	*PLEK2*	4.64	0.02
RHOC	25.22	0.70	*DKK3*	4.54	0.00
CCND1	21.20	0.05	*COL4A2*	4.36	0.00
NME1	19.50	0.18	*KRT5*	4.14	0.02
FSCN1	18.66	0.04	*MMP2*	4.10	0.02
CAV2	17.46	0.03	*MMP7*	4.08	0.00
EpCAM	17.20	0.02	*PTK2*	3.88	0.03
NME4	17.14	0.27	*GUSB*	3.48	0.32
VIM	16.50	13.90	*MAP1B*	3.48	0.00
ITGB1	15.68	0.80	*MCAM*	3.32	0.00
METAP2	13.76	0.52	*TIMP3*	3.30	0.00
F11R	12.84	0.09	*SERPINB5*	3.28	0.00
EGFR	12.72	0.02	*S100A4*	3.12	18.64
EWSR1	11.78	1.01	*SNAI2*	3.10	0.00
HPRT1	10.88	0.34	*HMBS*	2.92	0.07
PNN	10.82	0.77	*HTATIP2*	2.76	0.25
CTSL	10.54	0.11	*CTBP1*	2.68	0.23
KRAS	10.26	0.45	*TCF3*	2.68	0.08
FXYD5	9.46	5.07	*GEMIN2*	2.66	0.03
FGFBP1	9.40	0.00	*CAMK2N1*	2.64	0.00
CHD4	9.30	0.35	*SOX2*	2.64	0.00
DSP	8.62	0.03	*TGFB1*	2.60	0.74
DENR	8.14	0.35	*FGFR4*	2.60	0.00
AHNAK	7.64	1.87	*BMP7*	2.56	0.00
ECT2	7.18	0.02	*MMP14*	2.54	0.00
CD44	7.16	1.80	*ILK*	2.50	0.48
HRAS	6.86	0.05	*DESI1*	2.46	0.14
TFRC	6.62	0.07	*SSTR2*	2.44	0.00
ITGA6	6.34	0.12	*AKT1*	2.38	0.10
TGFB2	2.38	0.00	*VCAN*	0.00	2.18
LAMB1	2.36	0.00	*CST7*	0.00	2.02
TBP	2.30	0.15	*ZEB2*	0.00	0.77
FOXC2	2.22	0.00	*TGFBR2*	0.00	0.47

ETV4	2.10	0.00	*PECAM1*	0.00	0.41
VPS13A	2.02	0.17	*CXCR4*	0.00	0.41
TFPI2	1.98	0.00	*CASP8*	0.00	0.4
SMAD2	1.90	0.21	*RB1*	0.00	0.38
CD82	1.82	0.42	*MTSS1*	0.00	0.34
TIMP2	1.80	0.2	*RGS2*	0.00	0.32
MDM2	1.80	0.15	*PLAUR*	0.00	0.25
JAG1	1.78	0.00	*PTEN*	0.00	0.2
MTA2	1.76	0.16	*TIAM1*	0.00	0.12
CDH2	1.76	0.00	*TP53*	0.00	0.12
SYK	1.70	0.47	*SNAI3*	0.00	0.10
APC	1.70	0.15	*MYCL*	0.00	0.09
DSC2	1.68	0.02	*DAPK1*	0.00	0.09
ITGA5	1.64	0.11	*IL1B*	0.00	0.08
FAT1	1.60	0.00	*IL1RN*	0.00	0.08
TWIST1	1.50	0.00	*HPSE*	0.00	0.07
RBL2	1.46	0.74	*ZEB1*	0.00	0.07
CDH1	1.42	0.00	*SMAD4*	0.00	0.06
FLT4	1.42	0.00	*BAP1*	0.00	0.06
TCF4	1.38	0.50	*NOTCH1*	0.00	0.06
CCNE1	1.38	0.00	*RBL1*	0.00	0.05
GATA4	1.30	0.00	*MGAT5*	0.00	0.04
BMP1	1.22	0.00	*TCF20*	0.00	0.03
IPO8	1.20	0.09	*CXCR2*	0.00	0.03
KRT7	1.20	0.00	*NCAM1*	0.00	0.03
MET	1.18	0.00	*ERBB2*	0.00	0.02
CTNNB1	1.14	0.26	*SRC*	0.00	0.02
LYPD3	1.06	0.02	*GNG11*	0.00	0.02
NETO2	1.06	0.00	*MITF*	0.00	0.02
NF2	1.00	0.06	*HGF*	0.00	0.02
LEF1	0.98	0.71	*ITGAV*	0.00	0.02
RET	0.96	0.00	*SPARC*	0.00	0.02

Epithelial cells (n = 50) and PBMCs (n = 700) were enriched with *EpCAM* and *PTPRC* from the sequencing matrix, respectively. A total of 160 genes with positive reads either in KYSE270 cells or PBMCs from original 224 EMT and tumor metastasis genes are listed.

Table S8: Precise expression of *TWIST1* between the overexpression and knockdown groups in ESCC cell lines

ESCC Cells (Fold changes compared to control)	***TWIST1* Overexpression**		***TWIST1* Knockdown**	
	**RNA level (Log10)**	**Protein level**	**RNA level (Log10)**	**Protein level**
KYSE70	4.14	5.84	N/A	N/A
KYSE150	2.14	7.77	N/A	0.19
KYSE450	2.33	4.16	N/A	0.11

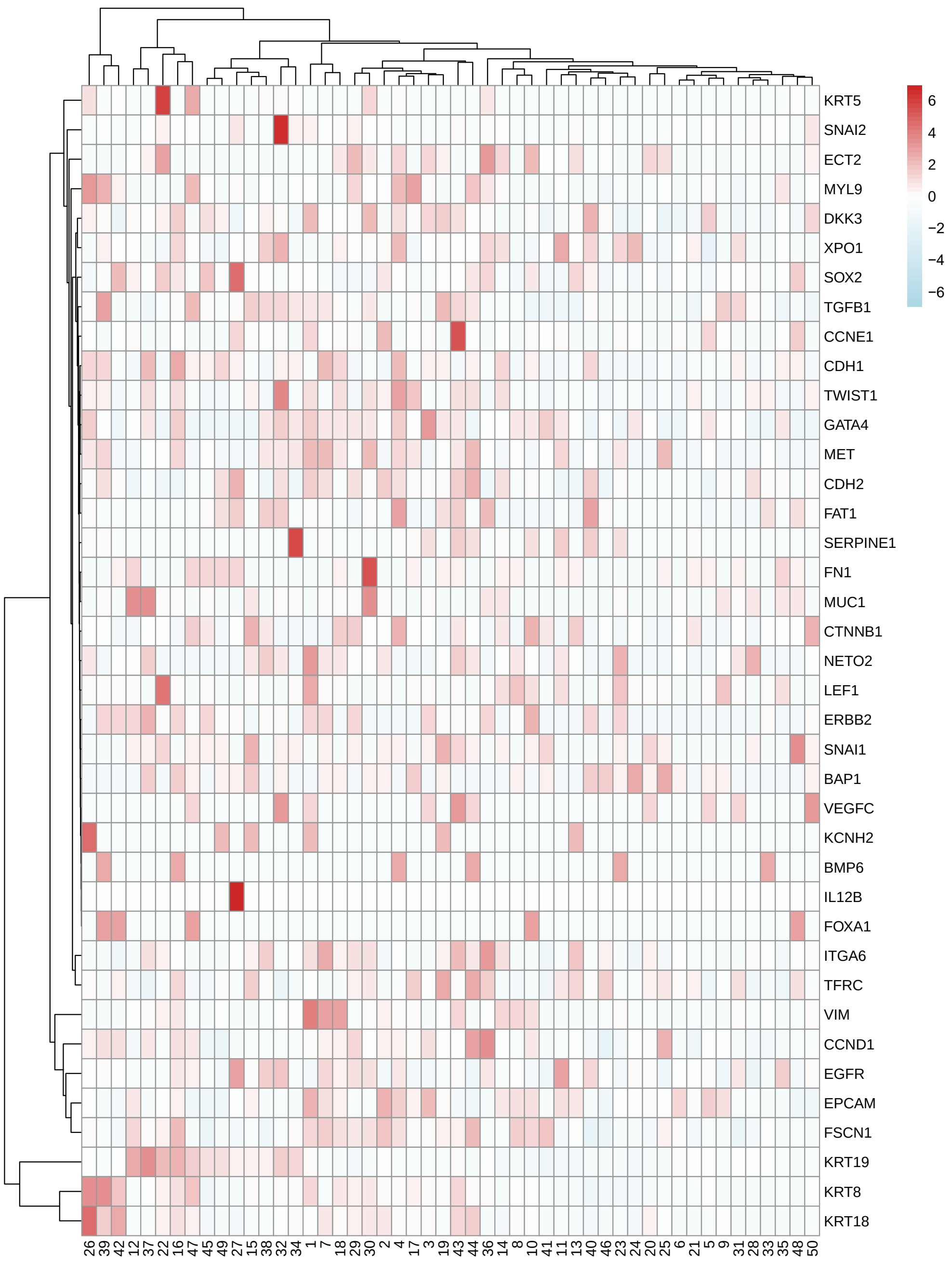

Figure S1: Gene expression heatmap of 40 putative CTC markers in 50 KYSE270 cells by 10x NGS sequencing

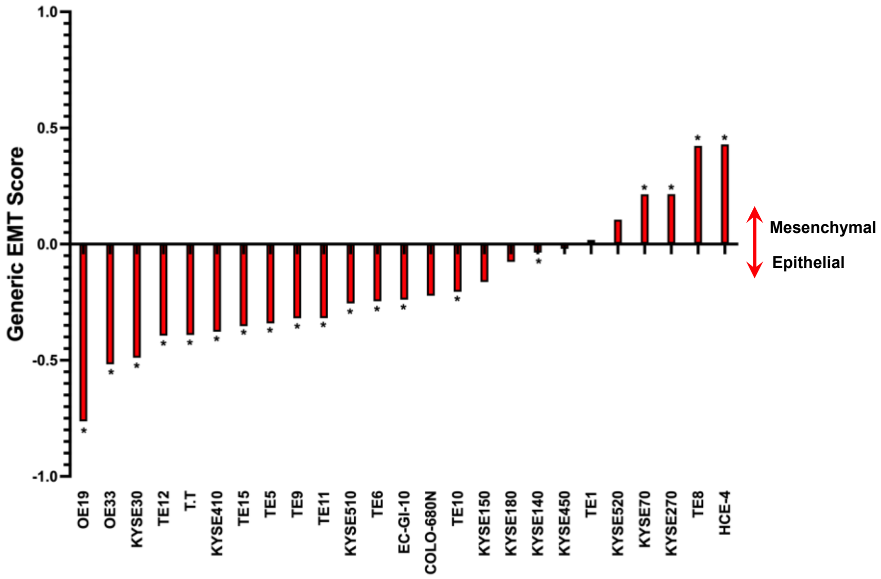

Figure S2: Scores of generic EMT signature of 25 esophageal carcinoma cell lines. Scores were computed based on the weighted sum of the significance analysis of microarray (SAM) and receiver operating characteristic (ROC) results from gene expression signature of 218 genes by Prof. J.P.Thiery and team. EMT score ranges from -1.0 (fully epithelial) to +1.0 (fully mesenchymal). Two-sample Kolmogorov-Smirnov test. *, p-value < 0.05.

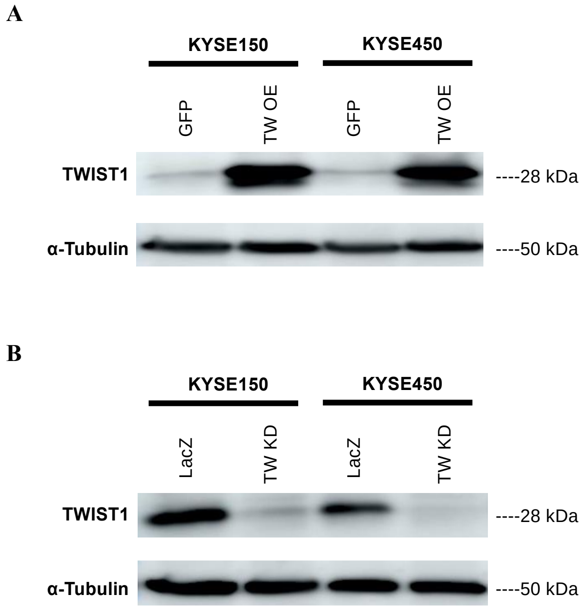

Figure S3: Validation of Lentiviral-mediated CRISPR activation and interference of *TWIST1* in representative ESCC cell lines by Western blotting. (**A**) GFP, negative control of CRISPR activation; α-Tubulin, loading control. (**B**) LacZ, negative control of CRISPR interference; α-Tubulin, loading control.

A

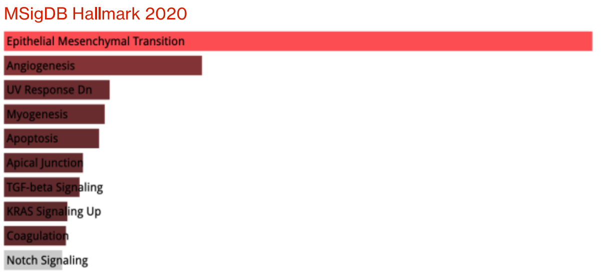

C

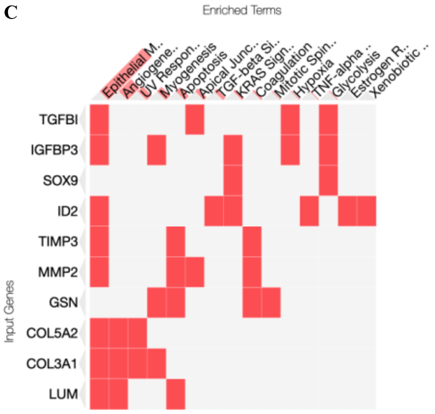

B

Index	Name	P-value	Adjusted p-value	Odds Ratio	Combined score
1	Epithelial Mesenchymal Transition	1.315e-12	4.077e-11	17.25	472.02
2	Angiogenesis	0.0007682	0.006084	18.62	133.53
3	UV Response Dn	0.0007850	0.006084	7.48	53.50
4	Myogenesis	0.0005070	0.006084	6.48	49.19
5	Apoptosis	0.001293	0.008015	6.66	44.30
6	Apical Junction	0.003324	0.01717	5.32	30.35
7	TGF-beta Signaling	0.02993	0.1102	7.79	27.33
8	KRAS Signaling Up	0.01807	0.08004	4.19	16.81
9	Coagulation	0.03200	0.1102	4.53	15.59
10	Notch Signaling	0.1483	0.2704	6.47	12.36

Figure S4: A comprehensive gene set enrichment analysis for top 100 up-regulated genes upon *TWIST1* overexpression was performed using EnrichR and the Molecular Signatures Database (MSigDB) Hallmark 2020. (**A**) Bar graphs (sorted by *p*-value ranking) represent the significance of that specific gene set using the MSigDB Hallmark 2020. The brighter the color, the more significant that term. (**B**) The table shows a raw view of the data and is sorted by *p*-value. Terms, *z*-score, odds ratio, and combined score are indicated. (**C**) The clustergrammer is sorted by combined scores (*p*-value and *z*-score) and shows heatmaps. Enriched terms are shown as columns and input genes as rows to understand the relationships between input genes and enriched terms. Cells in the matrix indicate whether a gene is associated with the indicated term.

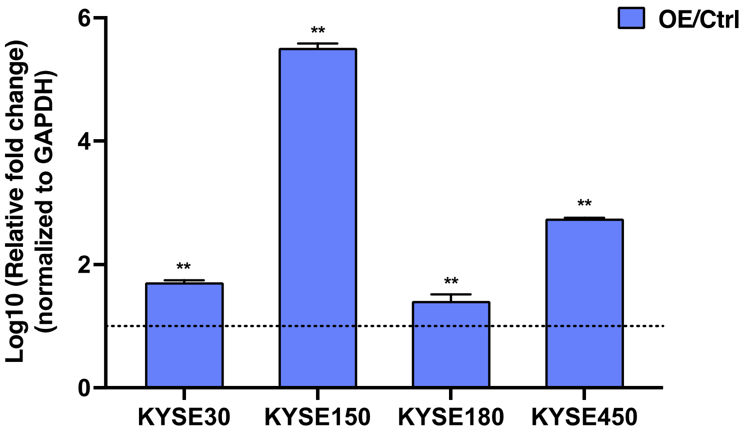

Figure S5: Validation of Lentiviral-mediated CRISPR activation of *TGFBI* in ESCC cell lines by RT-qPCR. RNA expression was quantified by RT-qPCR and normalized to *GAPDH*. Data is shown in Log10 (Fold change of *TGFBI* overexpression/control). Error bars represent standard error mean. The dotted line corresponds to a log10 fold change of 1. Ctrl: control; OE: overexpression. ** p-value < 0.01, Student's t test.

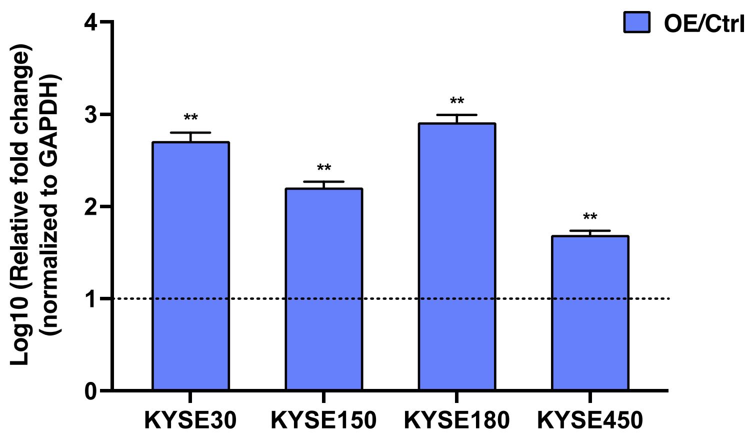

Figure S6: Validation of Lentiviral-mediated CRISPR activation of *ZEB1* in ESCC cell lines by RT-qPCR. RNA expression was quantified by RT-qPCR and normalized to *GAPDH*. Data is shown in Log10 (Fold change of *ZEB1* overexpression/control). Error bars represent standard error mean. The dotted line corresponds to a log10 fold change of 1. Ctrl: control; OE: Overexpression. ** p-value < 0.01, Student's t test.

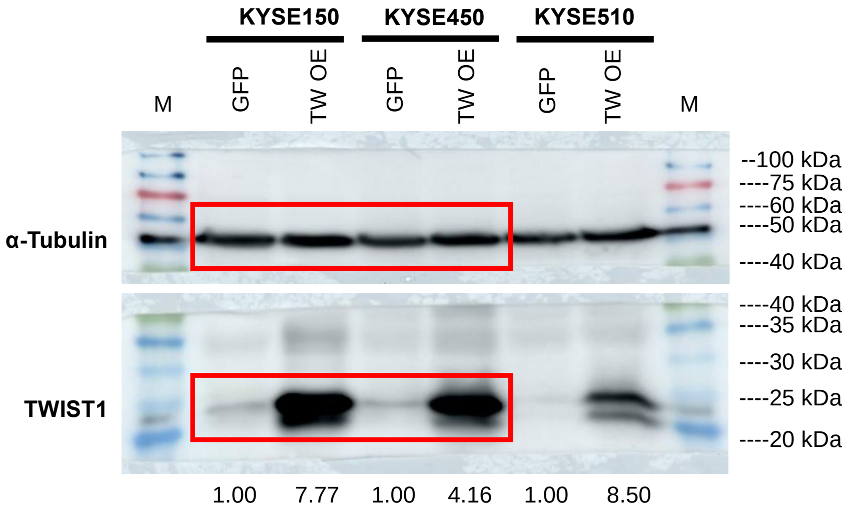

Figure S7. Uncropped Western blot figures for Figure S3A. Red box encircles the bands shown in the supplementary materials. Intensity ratio of each band normalized with loading control α-Tubulin and the GFP control cell line is shown underneath. M, molecular weight markers; GFP, negative control of CRISPR activation; α-Tubulin, loading control. Ctrl: Control; OE: Overexpression.

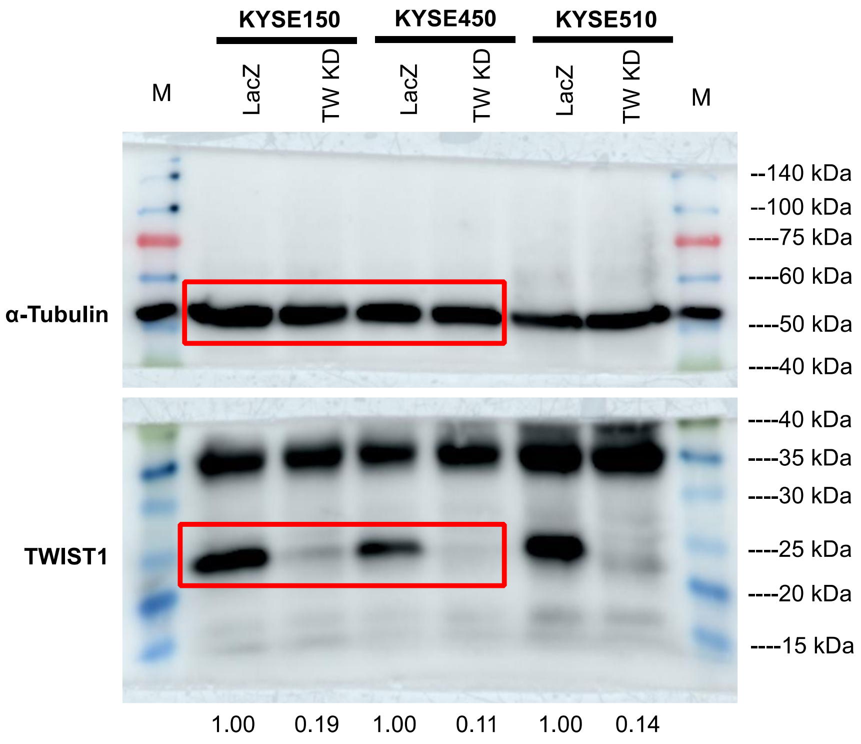

Figure S8. Uncropped Western blot figures for Figure S3B. Red box encircles the bands shown in the supplementary materials. Intensity ratio of each band normalized with loading control α-Tubulin and the LacZ control cell line is shown underneath. M, molecular weight markers; LacZ, negative control of CRISPR interference; α-Tubulin, loading control. Ctrl: Control; KD: Knockdown.
